# Case of psychological consultation and observation an adolescent with dissociative dysmnesia

**DOI:** 10.1192/j.eurpsy.2021.1671

**Published:** 2021-08-13

**Authors:** N. Zvereva, E. Zaytseva, M. Zvereva, A. Sinkevich

**Affiliations:** 1 Clinical Psychology, Federal State Budgetary Scientific Institution Mental Health Research Center; MSUPE, Moscow, Russian Federation; 2 Neuro- And Pathopsychology Of Development, MSUPE, Moscow, Russian Federation; 3 Clinical Psychology, Federal State Budgetary Scientific Institution Mental Health Research Center, Moscow, Russian Federation; 4 Project “close People”, Volunteers to help orphaned children Charity Foundation (BLAGOTVORITELNY FOND VOLONTERY V POMOSHCH DETYAM-SIROT), Moscow, Russian Federation

**Keywords:** adolescent, dissociative dysmnesia, Case study

## Abstract

**Introduction:**

Cases of memory loss are rare phenomena. We present a case-study of common psychological and psychotherapy observation 14,5 years old girl with dissociative dysmnesia during 2 years. Teenager grew up in a small town near Moscow, her parents were divorced. A girl and her elder brother stay with mother. One day in New Year Holiday in January she had come to take out the trash and did not return home. When mother and her friends had found girl on the other side of town, she did not remember anything about her life and family. Goal: description the case of long observing adolescent with dissociative dysmnesia

**Objectives:**

Girl age 14,5 y.o.

**Methods:**

Psychodiagnostics, Psychotherapy.

**Results:**

First period (1 months later initial episode) mother provided mane consultations to understand what has happened with daughter and what is illness. Psychiatrist made a diagnosis - dissociative dysmnesia, neurologists did not find any disturbances. Psychological diagnostics showed small gaps in knowledge, pronounced violations of autobiographical memory, decreased activity, and mood. Unusual results were obtained in projective drawing. She got to know her family and friends again, started an account on Internet, shares her stories. These 2 years she worked with individual psychotherapist with positive changes. In September 2020 she went to school, not yet caught up with program. She masters the guitar, continues to play in the theater studio, and makes plans for future.

**Conclusions:**

After 2 year observation and work autobiographical memory has not restored. Girl’s personality hasn’t changed, according to the family. Her mood and communication look better.

**Disclosure:**

No significant relationships.

